# Biologically Induced Deposition of Fine Suspended Particles by Filter-Feeding Bivalves in Land-Based Industrial Marine Aquaculture Wastewater

**DOI:** 10.1371/journal.pone.0107798

**Published:** 2014-09-24

**Authors:** Yi Zhou, Shaojun Zhang, Ying Liu, Hongsheng Yang

**Affiliations:** Key Laboratory of Marine Ecology and Environmental Sciences, Institute of Oceanology, Chinese Academy of Sciences, Qingdao, P. R. China; Centro de Investigacion Cientifica y Educacion Superior de Ensenada, Mexico

## Abstract

Industrial aquaculture wastewater contains large quantities of suspended particles that can be easily broken down physically. Introduction of macro-bio-filters, such as bivalve filter feeders, may offer the potential for treatment of fine suspended matter in industrial aquaculture wastewater. In this study, we employed two kinds of bivalve filter feeders, the Pacific oyster *Crassostrea gigas* and the blue mussel *Mytilus galloprovincialis*, to deposit suspended solids from marine fish aquaculture wastewater in flow-through systems. Results showed that the biodeposition rate of suspended particles by *C. gigas* (shell height: 8.67±0.99 cm) and *M. galloprovincialis* (shell height: 4.43±0.98 cm) was 77.84±7.77 and 6.37±0.67 mg ind^−1^•d^−1^, respectively. The total solid suspension (TSS) deposition rates of oyster and mussel treatments were 3.73±0.27 and 2.76±0.20 times higher than that of the control treatment without bivalves, respectively. The TSS deposition rates of bivalve treatments were significantly higher than the natural sedimentation rate of the control treatment (*P*<0.001). Furthermore, organic matter and C, N in the sediments of bivalve treatments were significantly lower than those in the sediments of the control (*P*<0.05). It was suggested that the filter feeders *C. gigas* and *M. galloprovincialis* had considerable potential to filter and accelerate the deposition of suspended particles from industrial aquaculture wastewater, and simultaneously yield value-added biological products.

## Introduction

Land-based flow-through (FT) aquaculture production systems are being widely used around the world. The N and P nutrient discharge from FT aquaculture should be regulated to mitigate effluent nutrient contributions to the receiving waters, which may lead to degradation and eutrophication [1]. Land-based intensive marine recirculating aquaculture system (RAS), as the newest form of fish farming production system, has been rapidly developing in the past decade [2]. RAS is typically an indoor system that allows farmers to control environmental conditions throughout the year. Suspended waste solids, including uneaten feed and fish feces, have to be removed as quickly as possible to prevent their accumulation to unsafe levels within the RAS. If left in the system, the suspended solids could generate additional oxygen demand and ammonia nitrogen as a result of bacterial decomposition. Thus, removal of suspended solids is one of the critical processes in RAS [3]–[5]. Large particles are easily removed from industrial aquaculture wastewater via filter techniques or settling tanks, while small particles are too small to be retained by traditional filter techniques, and they have a settling velocity too small to be trapped in sedimentation basins. In addition to the general mechanical filtration that removes larger suspended solids, methods such as microfiltration or sieve-bend screen have been applied to remove the suspended solids of medium particle size. Although the removal accuracy of suspended solids has generally ranged from 100 to 200 µm, the removal rate of suspended solids of smaller particle size less than 100 µm has been relatively low. Chen et al. [3] found that, using conventional filter techniques, it is difficult to remove particulate matters with a diameter less than 30 µm, which account for 80–90% of the total mass of particulate matters in the water body of high-density RAS. In particular, the feces in the discharged water from the finfish culture of *Cynoglossus semilaevis* Günther easily breaks down into flocculent suspended solids which are difficult to be precipitated and removed. This not only increases the processing load of the water treatment unit of the RAS, but also reduces the contact area of the biofilm with the dissolved pollutants in wastewater and influences the processing efficiency of the biofilm due to the adhesion of fine suspended solids [3]–[5].

Filter-feeding bivalves, such as oysters and mussels, have strong water-filtration ability. They are able to filter a large number of fine particulate matters, including phytoplanktons, zooplanktons, microorganisms (e.g. bacteria [6]), and other particulate organic debris [7], and cause sedimentation of the suspended solids in the form of feces and pseudofeces. This process is known as biodeposition [8]–[9]. Furthermore, the organic components in the suspended matters can be assimilated and utilized by filter-feeding bivalves. Many studies have shown that mussels, oysters, clams, and other filter-feeding bivalves are very important components of a healthy coastal ecosystem, and act as key species and functional group [10]–[11]. These bivalves are not only economically vital, but also ecologically important. They significantly enhance the coupling between water layer and benthos through physiological and ecological processes such as filter-feeding and biodeposition, and play an important role in material cycling and energy flow in coastal ecosystems [7], [9], [12]–[13]. Recently, the filter-feeding capacity of bivalves has been proposed to have the potential to purify organic effluents released from open-water fish farms and shrimp ponds; in the Integrated Multi-Trophic Aquaculture (IMTA), the filter-feeding bivalves not only remove the suspended particulate matters in the water body, but also transform these wastes into economically valuable products [14]. Nowadays, there is an increasing interest in the IMTA as a sustainable approach to fed aquaculture (e.g. finfish, shrimp). In the IMTA, fed aquaculture is combined with inorganic (nutrient) extractive aquaculture (e.g. seaweed [14]–[16]), suspended organic extractive aquaculture (e.g. filter-feeding bivalves [17]–[18]), and deposited organic extractive aquaculture (e.g. sea cucumber [19]–[20]) to create balanced systems that provide environment bioremediation, mutual benefits to cocultured organisms, and economic diversification by producing other value-added products [14], [21].

For the occurrence of large quantities of fine suspended particles in industrial aquaculture wastewater, filter feeders may offer an approach for treatment of those suspended matter. Surprisingly, little work has been done on removal of fine suspended particles from industrial marine aquaculture wastewater using macro-bio-filters. Our previous work showed that suspension-cultured filter-feeding bivalves (*Chlamys farreri*) can greatly increase the deposition of suspended particulate matter in coastal eutrophic waters [9], [13]. In the present study, our hypothesis was that bivalve filter feeders could filter and accelerate the deposition of suspended particles from industrial aquaculture wastewater. We tested the use of two filter-feeding bivalve species (the Pacific oyster *Crassostrea gigas* and the blue mussel *Mytilus galloprovincialis*) with strong adaptability and water-filtration ability to remove small suspended particles from the marine RAS. The treatment potential of the two bivalves to deposit suspended particulate matters in saline wastewater from the RAS was experimentally determined.

## Material and Methods

### Ethics Statement

The collecting of the bivalves (*Crassostrea gigas* and *Mytilus galloprovincialis*) used in this experiment from an adjacent coastal area (Jiaozhou) was permitted by Benshan Wang, manager of that area. Ethical approval was not required for this study because no endangered animals were involved. However, specimen collection and maintenance were performed in strict accordance with the recommendations of Animal Care Quality Assurance in China.

#### 2.1. Experimental design

**Figure 1:**
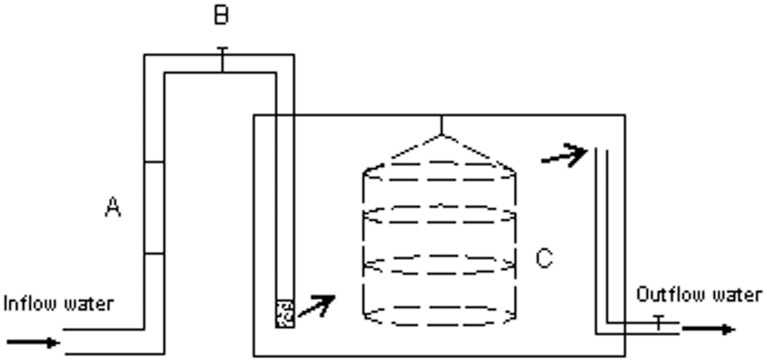
Schematic diagram of individual experimental biotanks. Each biotank measured 75×50×50 cm in size. A, Flowmeter; B, Control valve; C, Lantern net deployed in a biotank for bivalve culture.

The FT experiment included three treatments, i.e. oyster treatment, mussel treatment, and control treatment. Each treatment consisted of five plastic biotanks (Figure 1), with each biotank measuring 75×50×50 cm in size; and a total of 15 plastic biotanks were used. In each bivalve treatment, the experimental bivalves were evenly placed in a four-tray lantern net, while in the control treatment, only an empty lantern net was placed. In each biotank, the bottom of the inflow pipe was sealed, and small holes were perforated at approximately 10 cm on the lower part of the pipe to allow advection of water into the biotank.

The experiment was conducted at a land-based aquaculture site (Haifa Company, Tianjin, norh China) for the finfish *Cynoglossus semilaevis* Günther. The fish (40–50 cm in body length, 550–900 g ind^−1^ in wet weight) was intensively cultured in a density of 25 ind m^2^ in 38-m^2^ culture tanks (0.8 m in depth). During the culture, formula feed with an amount of 1–2% that of wet weight of fish was fed twice a day. The bivalves (*Crassostrea gigas* and *Mytilus galloprovincialis*) used in the experiments were collected on 18 November 2012 from the adjacent coastal area. The experimental wastewater was the discharged water from the intensive culture of *C. semilaevis*, which was filtered through sieve-bend screen. The water temperature was 20.1–24.6°C, dissolved oxygen (DO) was 6.04–7.83 mg L^−1^, and pH was 7.60–7.81. The experimental wastewater was pumped into an elevated cistern and then piped into the systems; the volume of each experimental biotank was 150 L. The experiment was conducted from 26 November 2012 and lasted for 7 days. The flow rate of the inflow water was 80 L h^−1^, and the stocking densities in each biotank were 13 individuals with total wet weight 1038.96±14.01 g for oysters and 110 ind with total wet weight 765.61±24.05 g for mussels. The total dry tissue weight of bivalves in each oyster biotank and mussel biotank was 27.29±1.10 g, and 22.01±1.73 g (Table 1), respectively. Before the initiation of the experiment, the bivalves were acclimated in the experimental systems for one week.

**Table 1:** Biological parameters of bivalves used in the experimental systems.

Treatments	Shell height (cm)	Total wet weight (g)	Total dry tissue weight (g)	Individual dry tissue weight (g•ind^−1^)
Oyster	8.67±0.99	1038.96±14.01	27.29±1.10	2.10±0.12
Mussel	4.43±0.98	765.61±24.05	22.01±1.73	0.20±0.01

#### 2.2. Sampling and determination

At the end of the experiment, the inflow water was stopped and the shellfishes were taken out. The biotank was left to stand for 5 h. The sediments were collected using siphonage, desalted and baked at 60°C, and weighed to obtain the dry weights. The contents of organic matter (OM), organic carbon (OC), organic nitrogen (ON), inorganic phosphorus (IP), organic phosphorus (OP), and total phosphorus (TP) in the sediments were measured. The OM was expressed as the difference in weight before and after ashing at 500°C for 3 h. After the sediments were decarbonated using acid mist of concentrated hydrochloric acid for 5 h, the OC and ON were measured using a CHN elemental analyzer. The IP, OP, and TP in the sediments were measured using the ashing method [22]. The shell heights and wet weights of the oysters and mussels in each cage were measured.

The formula for determining the sedimentation rate (mg·d^−1^) of suspended solids in the system was as follows: 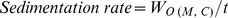
where W_O (M, C)_ is the dry weight (mg) of total sediments collected from the bottom of the single biotank of oyster (or mussel/control) treatment, and t is the experiment duration (days). The biodeposition rates (BDR; mg·ind^−1^·d^−1^) of the suspended solids by the two kinds of bivalves were calculated using the following formula:
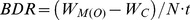
where W_M (O)_ is the dry weight (mg) of the total sediments collected from the bottom of the biotank of oyster (or mussel) treatment, W_C_ is the dry weight (mg) of the total sediments collected from the bottom of the biotank in the control, t is the experiment duration (days), and N is the number of bivalves used in each treatment.

During the experiment, the total suspended solids (TSS; mg L^−1^) in the inflow and outflow water of the system were determined 3 times every day. For TSS determination, 2 L of water sample was filtered through a pre-weighed mixed fiber membrane with a diameter of 1.0 µm. After filtration, the filter was rinsed with 100 mL of distilled water to remove the salts, dried at 60°C for 48 h, and weighed.

In the present study, results are presented as mean ± SD. The differences between the treatments were tested using one-way analysis of variance (ANOVA). Prior to analysis, data were examined for homogeneity of variances using Levene’s tests. The differences were considered significant at a probability level of 0.05. Statistics were performed using the software SPSS 16.0.

## Results

The concentration of the total suspended solids (TSS; mg L^−1^) in inflow water in the experimental system was 3.57±2.37 mg L^−1^. The TSS sedimentation rates of the bivalve treatments were significantly higher than the natural sedimentation rate of the control treatment (*df* = 9, *F* = 331.0, *P*<0.001; *df* = 9, *F* = 166.6, *P*<0.001). The TSS sedimentation rates of oyster and mussel treatments were 3.73±0.27 and 2.76±0.20 times higher than that of the control treatment, respectively. The biodeposition rates of the oyster *C. gigas* (shell height: 8.67±0.99 cm; Table 1) and the mussel *M. galloprovincialis* (shell height: 4.43±0.98 cm) were 77.84±7.77 and 6.37±0.67 mg·ind^−1^·d^−1^, respectively (Table 2). Compared with control treatment, TSS in the outflow water in oyster and mussel treatments was averagely reduced 21.3±3.7% and 14.1±2.6%, respectively.

**Table 2:** Biological filter-removing rates of suspended particles in aquaculture wastewater by experimental bivalves.

Treatments	Sedimentation rates (mg•d^−1^)	Ratios of sedimentation rates(Bivalve/Control)	Biodeposition rates (mg•ind^−1^•d^−1^)	Percentage of TSS reduced (%)
Oyster	1381.9±101.0^a^	3.73±0.27^a^	77.84±7.77^a^	21.3±3.7^a^
Mussel	1020.7±75.8^b^	2.76±0.20^b^	6.37±0.67^b^	14.1±2.6^b^
Control	370.0±79.3^c^	-	-	-

Sedimentation rates (mg•d^−1^): the sedimentation rate of suspended solids in the FT system; Ratios of sedimentation rates: ratios of the sedimentation rate of suspended solids in a bivalve treatment to that in the control (without bivalves); Percentage of TSS reduced (%): percentage of TSS reduced in the outflow water in oyster or mussel treatment, compared with control treatment without bivalves. Values are given as means±SD. Values with different superscripted letters in the same column are significantly different from each other (*P*<0.05).

**Figure 2:**
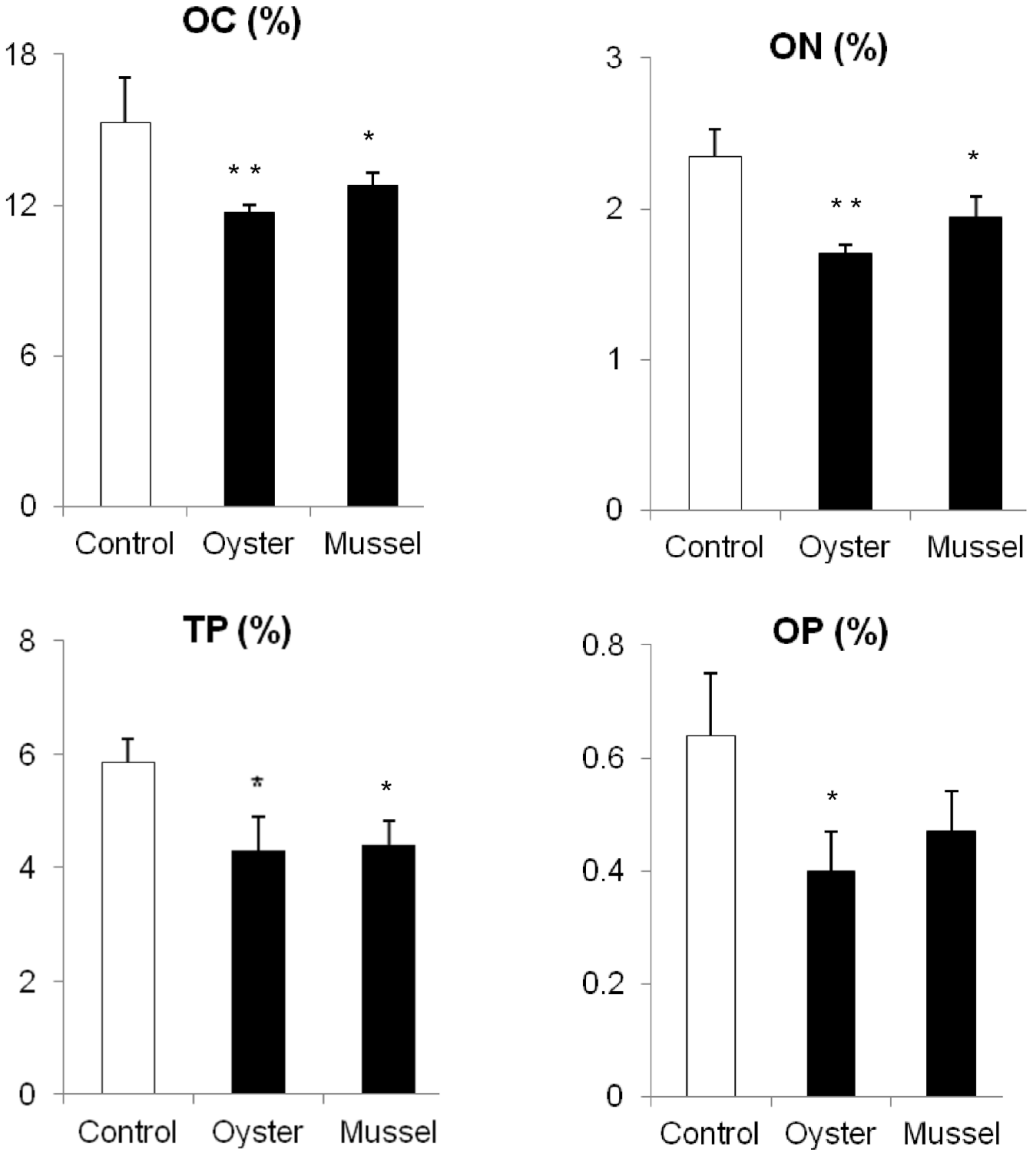
Comparisons in C, N, and P in the deposited sediments between a bivalve (oyster or mussel) treatment and the control (without bivalves). OC, organic carbon; ON, organic nitrogen; TP, total phosphorus; OP, organic phosphorus. *, significant difference between control and bivalve treatment (*P*<0.05); **, *P*<0.01. Values are means ± SD.

Table 3 shows the contents of organic matter (OM) and C, N, and P in the sediments of bivalve and control treatments. The OM content in the sediments of oyster treatment was 29.46±0.45%, which was significantly lower than that in the sediments of the control (38.44±3.19%; *P*<0.001). Furthermore, the contents of C, N, and P in the sediments of oyster treatment were 11.72±0.29, 1.70±0.06, and 4.30±0.06%, respectively, while those in the sediments of control treatment were 15.29±1.79, 2.34±0.19, and 5.85±0.43%, respectively. These results showed that the OM and C, N, and P contents were significantly lower in the sediments of oyster treatment than in the sediments of the control treatment (Figure 2). Also, there were significant differences in the OM and C, N, and P contents in the sediments of the mussel and control treatments (all *P*<0.05).

**Table 3:** Comparisons in chemical compositions of sediments between bivalve (oyster or mussel) treatment and the control (without bivalves) in the FT experiments.

Treatments	OM (%)	OC (%)	ON (%)	TP (%)	OP (%)	C/N	C/OP
Control	38.44^a^	15.29^a^	2.34^a^	5.85^a^	0.64^a^	7.65^a^	58.0^c^
(±SD)	(3.19)	(1.79)	(0.19)	(0.43)	(0.11)	(0.28)	(6.4)
Oyster	29.46^c^	11.72^c^	1.70^c^	4.30^bc^	0.40^b^	8.04^a^	76.1^a^
(±SD)	(0.45)	(0.29)	(0.06)	(0.06)	(0.07)	(0.24)	(10.0)
Mussel	32.70^b^	12.79^b^	1.94^b^	4.40^b^	0.47^ab^	7.69^a^	71.9^ab^
(±SD)	(1.63)	(0.50)	(0.14)	(0.13)	(0.07)	(0.28)	(9.3)

Values are given as means±SD. OM, organic matter; OC, organic carbon; ON, organic nitrogen; TP, total phosphorus; OP, organic phosphorus. Values with different superscripted letters in the same column are significantly different from each other (P<0.05).

## Discussion

In recent years, application of filter-feeding bivalves for the removal of suspended particulate matter from the fish and shrimp aquaculture system in coastal waters has been reported [14]. In these systems, the food sources of bivalves include phytoplankton, uneaten feed, and fish feces. However, there have been no reports on the application of the macro-bio-filters for the removal of suspended particles from land-based industrial aquaculture systems, which are difficult to be removed using conventional mechanical filtration. In the present study, we conducted the FT experiment and found that the bivalve filter feeders, *Crassostrea gigas* and *Mytilus galloprovincialis*, had marked potential to deposit suspended solids in discharged wastewater from fish aquaculture.

Under the FT condition, the TSS sedimentation rates of the oyster and mussel treatments were 1381.9±101.1 mg d^−1^, and 1020.7±75.8 mg d^−1^, respectively, with the former significantly higher than the latter (Table 2). The difference might be due to the higher total dry tissue biomass (27.29±1.10 g) in each oyster treatment than that in each mussel treatment (22.01±1.73 g; Table 1). Also, dry tissue weight of bivalves (2.10±0.12 g ind^−1^) in oyster treatments was almost ten times higher than that in oyster treatments (0.20±0.01 g ind^−1^; Table 1); this might explain the much higher biodeposition rate of oysters (77.84±7.77mg·ind^−1^·d^−1^) than that of mussels (6.37±0.67 mg·ind^−1^·d^−1^; Table 2) in this experiment.

Many studies have shown that the filter-feeding bivalves have strong water-filtration abilities and can exert profound impact on the suspended particulate matter in coastal waters. Hatcher et al. [23] determined the deposition rates in the Upper South Cove mussel aquaculture area in Canada and compared the results with those in the neighboring non-culture areas. They found that the amount of deposited materials in the former area was more than two times higher than that in the latter area. In many bivalve aquaculture areas, the biodeposition rate has been noted to be very impressive. For example, in the oyster aquaculture area in Hiroshima bay (Japan), 420,000 oysters could produce 16 metric tons of feces and pseudofeces in a 9-month culture period [24]. Since faeces and pseudofaeces are voided as mucus-bound aggregates, they are larger and more prone to sedimentation than the nonaggregated particles from which they are formed, and deposit at rates up to 40 times that of nonaggregated particles [25]–[27]. In a mussel farm covering an area of 1500 m^2^ in Sweden, the biodeposition of dry matter was estimated to about 10 metric tons, and sediment under the rafts would accumulate to about 10 cm during a farming period of 1.5–2 years, and mussel farming was suggested to be a potential way to improve water quality in a eutrophied system [28]–[29].

The present study conducted in a land-based industrial aquaculture site showed that under the FT condition, the bivalves, *C. gigas* and *M. galloprovincialis*, both exhibited high suspended solid filtration rate in the effluent from *C. semilaevis* culture. The average biodeposition rate of individual oysters and mussels were 77.84±7.77 and 6.37±0.67 mg·ind^−1^·d^−1^, respectively. By contrast, the biodeposition rate of *C. gigas* (shell length: 95–110 mm) in natural aquaculture conditions in the Xuanmen port (north China) was 37.5–83.7 mg·ind^−1^·d^−1^ [30]. The main factors affecting biodeposition rates of bivalves include body size, water temperature, seston concentration, and composition [9], [13]. The wastewater used in this study was the effluent from the *C. semilaevis* culture. The main components of the suspended solids were feed wastage and fish feces. In coastal fish-bivalve polyculture, the particulate organic matter from feed wastage and fish feces has been shown to be the potential food source for filter-feeding bivalves [17], [31]–[32]. In the present study, the OM and C, N, and P contents in the sediments of bivalve treatments were significantly lower than those in the sediments of the control (Table 3; Figure 2), indicating that the bivalves could assimilate the organic matter in the suspended solids in aquaculture wastewater.

In summary, considering the fact that large quantities of fish suspended solids in discharged water from intensive finfish culture (*C. semilaevis*) are difficult to remove using conventional mechanical filtration, therefore we tried bivalve filter feeders (oysters and mussels) in this study to perform biological filtration and deposition. The present study demonstrated that both bivalve species had strong biological treatment potential for accelerating the deposition of suspended solids in effluent discharged from fish culture tanks. The biologically induced deposition of suspended solids in industrial mariculture wastewater by filter-feeding bivalves might achieve the goals of removing the suspended solids as well as transforming the aquaculture wastes into biological resources. Before promoting the use of these bivalves as biofilters, it is necessary to evaluate the possible content of pathogens in them. It is suggested that future work should be focused on developing an effective way to employ bivalve filter feeders to remove fish suspended solids in land-based industrial marine aquaculture wastewater.
